# Contamination of multi dose eyedrops in the intra and perioperative context

**DOI:** 10.1038/s41598-021-99892-8

**Published:** 2021-10-13

**Authors:** Tristan Daehn, Andreas Schneider, Johannes Knobloch, Olaf J. C. Hellwinkel, Martin Stephan Spitzer, Robert Kromer

**Affiliations:** 1 Department of Ophthalmology, University Medical Center Hamburg-Eppendorf, Martinistraße 52, 20246 Hamburg, Germany; 2 Department of Medical Microbiology, Virology and Hygiene, University Medical Center Hamburg-Eppendorf, Martinistraße 52, 20246 Hamburg, Germany; 3 Department of Forensic Medicine, University Medical Center Hamburg-Eppendorf, Martinistraße 52, 20246 Hamburg, Germany

**Keywords:** Medical research, Risk factors

## Abstract

In this study, we examined the rate of contamination of multi-dose ophthalmic solutions in the operating theatre and the underlying risk for infection by examining the microbiological load on the tips of the dispenser bottles. A total of 245 samples of eye drop bottles were collected and analysed between June 2018 and January 2019. All were collected in the operating theatre of the University Eye Hospital Hamburg-Eppendorf. Contamination of the dropper tip occurred in 2% of the samples. Although the prevalence of contamination was low, the results of this study reveal the possibility of contamination of multi-dose eyedrops even when used by health care professionals in the controlled environment of an operating theatre. Following these results, we recommend the use of single-dose eyedrops in the pre- and intraoperative context.

## Introduction

Endophthalmitis is a rare but devastating eye infection that may lead to irreversible blindness in the infected eye within hours or days of symptom onset^1^. The disease can broadly be divided into exogenous and endogenous endophthalmitis; the majority of cases are exogenous^1^. Postoperative endophthalmitis accounts for 40% to 80% of endophthalmitis cases seen at centres in Brazil, England, Israel, Iran, India, Australia, and South Korea^2,3^. While the primary source of bacteria in culture-positive cases of endophthalmitis is presumed to be the ocular surface and adnexa of the patient^4^, further measures are nevertheless necessary to decrease other potential triggers.

Multi-dose ophthalmic solutions may become contaminated with microorganisms during repeated use, with studies identifying contamination rates varying from 0.07 to 70%^5–7^. The pathogens detected are mainly those of the skin flora and the environment; however, *Staphylococcus aureus*, *Pseudomonas aeruginosa*, and *Proteus mirabilis* have also been detected with lower frequency^8–11^. While inflammation due to contamination seems to be infrequent and preservatives should inactivate pathogens, cases of keratitis^12^, conjunctivitis^10,13^, and endophthalmitis^14^ likely caused by contamination have been reported. Contamination is most commonly attributed to improper use^15^. Nentwich et al. studied the contamination of ophthalmic solutions in different environments inside an ophthalmology clinic but was not able to detect contamination of ophthalmic solutions inside the operating theatre using a limited sample size, while Teuchner et al. detected a small amount of contamination^5,15^.

In Germany the Commission for Hospital Hygiene and Infection Prevention at the Robert Koch Institute (KRINKO) draws up recommendations for the prevention of nosocomial infections as well as on operational-organizational and structural–functional measures of hygiene in hospitals and other medical facilities. The implementation of these measures in accordance with the Infection Protection Act (IfSG) is the responsibility of the hospital's own hygiene commission. As far as the authors are aware there are no specific guidelines regarding the differences of multi-dose or single-dose eyedrops.

Therefore the identification of potential hazards in the daily routine is essential to improve the medical care.

This study aimed to address the above-mentioned uncertainty of potential contamination of multi-dose ophthalmic solutions in the operating theatre and the underlying risk for infections by examining the microbiological load on the tips of the dispenser bottles.

## Methods

### Ophthalmic medications

Swabs of multi-dose ophthalmic solutions were collected every Friday during a 6-month period from the operating room of the Department of Ophthalmology at the University Medical Center Hamburg-Eppendorf. These eyedrops were only used within the respective operating theatre in accordance to the user manual*.* The date of expiry was checked on a daily base*.* They were applied to patients intraoperatively after patients were disinfected using an antiseptic (Iodine or polyhexanide) only by trained operating room nurses or ophthalmic surgeons. There were no restrictions regarding the multi-dose solutions that could be included in the study.

### Sample collection

**Figure 1:**
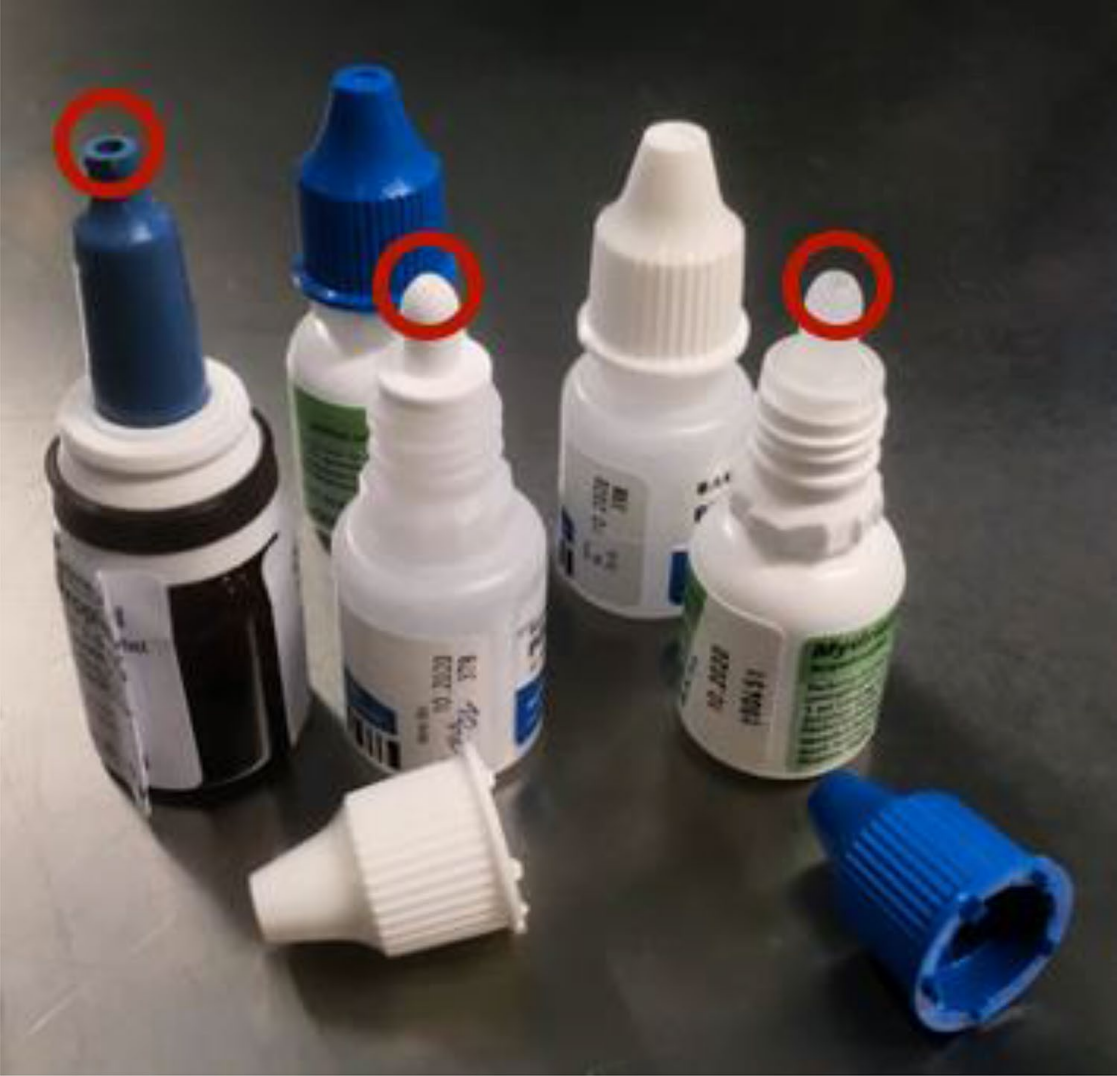
Examples of opened multi-dose eyedrop bottles with marked (red circle) tip of the bottle where swabs were taken (by Robert Kromer, 2020).

Samples were taken from the tips of the bottles (Fig. 1), as this area has been demonstrated to be contaminated most frequently^5,8–10,14,16^. The caps of the bottles were removed and the tips were smeared with ESwab (Copan Italia SpA), a multipurpose collection and transport system that maintains the viability of aerobic, anaerobic, and fastidious bacteria for up to 48 h at room temperature. Neither length of use before discard, number of patient use nor time to date of expiry were recorded.

### Incubation and identification of microorganisms

The Swabs were vortexed and the media samples were moved to plates with BD BBL Thioglycollate Medium enriched with vitamin K1 and hemin, a general-purpose medium for the cultivation of fastidious and non-fastidious microorganisms. All plates were incubated at 37 °C under aerobic conditions for 48 h and were observed for bacterial or fungal growth. Any visible growth was considered potential contamination and examined whether bacteria could be isolated. If colony-forming units were detected, the probes were sent to the Department of Medical Microbiology, Virology and Hygiene at the University Medical Center Hamburg-Eppendorf. The identification of bacteria species was performed using matrix-assisted laser desorption-ionization time of flight (MALDI-TOF) mass spectrometry fingerprinting described in detail elsewhere^17^.

### Ethical statements and institutional guidelines

We did not perform experiments on humans and/or used human tissue. The study was performed in accordance with the Declaration of Helsinki and the relevant national and institutional guidelines and regulations for publication. No approval by the ethics committee was required as no patient or staff data were recorded.

## Results

A total of 245 samples of multi-dose eyedrop solutions were collected and analysed from June 2018 until January 2019. Contamination of the dropper tip occurred in 2.0% of the samples (n = 5; Table 1). Four positive samples used benzalkoniumchloride as preservative one used Phenylmercurinitrate. Contamination was independent of the type of cap an closure (Table 2). Gram-positive organisms were cultivated from all five contaminated samples. *Staphylococcus hominis* was the most common bacteria and occurred in 1.2% of all samples. All identified organisms were part of the normal skin flora. None of the samples grew more than one type of bacterium. No fungal contamination was found in any specimen.

**Table 1:** Overview of contaminated multi-dose eyedrop bottles with respective eye medication, bottle size, preservative, and microorganism identified.

Number	Eye medication	Bottle size	Preservative	Microorganism identified
1	Oxybuprocain hydrochloride 1%	10 ml	Benzalkonium chloride	*Staphylococcus hominis*
2	Oxybuprocain hydrochloride 1%	10 ml	Benzalkonium chloride	*Staphylococcus hominis*
3	Neosynephrin-POS® 10%	10 ml	Benzalkonium chloride	*Staphylococcus capitis*
4	Neosynephrin-POS® 10%	10 ml	Benzalkonium chloride	*Staphylococcus epidermidis*
5	Mydriaticum	10 ml	Phenylmercurinitrate	*Staphylococcus hominis*

**Table 2:** Overview of examined multi-dose eyedrop bottles with respective eye medication, bottle size, preservative, type of cap and closure, and action and release mechanism.

Eye medication	Bottle size	Preservative	Type of cap and closure	Action and release mechanism
Oxybuprocain hydrochloride 1%	10 ml	Benzalkonium chloride	pull–push cap	dropper tip plug
Neosynephrin-POS® 10%	10 ml	Benzalkonium chloride	twist top cap	dropper tip plug
Mydriaticum	10 ml	Phenylmercurinitrate	twist top cap	dropper tip plug

## Discussion

In this study, we showed that there is a potential risk of contaminating eyedrops even if they are only used by health care professionals in an operating theatre.

Previous studies have shown that ophthalmic solutions may become contaminated with microorganisms during repeated use^5,8–10,14,16,18^. In these studies, the percentage of contamination has shown enormous variation, from 0.7% up to 70%^5–7^.

As previously demonstrated by Teuchner et. al percentage of contamination is significantly higher in the eye drops applied by patients (24,4%), used in the ward (19,5%), and in the outpatient unit (17,1%) compared with that in operating room (5%)^15^. This may be due to (1) shorter duration of use, (2) more restrictive hygienic provisons and, (3) higher room cleanliness and therefore lower bacterial load of the environment. Notably the aim of this study was an analysis of bacterial contamination of multi-dose eye drops under high hygienic standards and therefore explaining the lower percentage of 2%.

It is generally accepted that the rate of contamination increases with the time of use for application^6,8,19^, whereby improper use is seen as the main source of contamination^10^.

Nevertheless Fazeli et al., for example observed significant differences between eye drops which were used for seven days compared with drops that were used for one day. There were no significant differences between day 1, 2 and 4^6^. Many such studies were conducted in outpatient departments rather than operating theatre environment. In this case, none of the bottles had been in use for longer than 5 days, and the eyedrops had only been applied by health care professionals. Nevertheless, contamination of the bottle tips occurred in 2% of the cases. All detected bacteria were staphylococci and common residents of the skin flora^20^.

As shown by Hassan et al., coagulase-negative staphylococci are the most common pathogens causing endophthalmitis^22,22^, and ocular medication can be a possible source^12^. Consistent with previous studies, human pathogens not belonging to the environmental, conjunctival or skin flora were not detected in our investigation^5,6,10,19^.

According to previous studies, the rate of contamination on the tip of the bottle is higher than detectable contamination in the eyedrops^15^. Contamination of the fluid inside the bottle was mostly due to open storage (without the cap placed on the bottle), enabling bacteria to enter^15^. We exclude this kind of contamination in our operating theatre because, according to the hygienic standards, eyedrops must be closed immediately after usage.

There were clear limitations in this study. Neither length of use before discard, number of patient use nor the time to the expiry fate were recorded. However eyedrops were applied by the nurse within the respective operating theatre. These eyedrops were discarded at the end of the working week. Therefore we are not able to give a statement on time-dependent contamination (in the range of more than 5 days). Even so this is one of the biggest sample sizes in the literature, it was not possible to link contamination to a specific use of preservative or type of cap and closure.

Although it seems that single dose eye drops offer several advantages, the following aspects must be considered: (1) higher costs, (2) higher amount of plastic waste and (c) availability of pharmaceutical ingredients as single dose eye drops. Also it is not clear if there might occur bacterial contamination on the tips of single dose eye drops after due to the storage and opening procedure and therefore could also show an significant amount of contamination. Further studies should focus on these open questions and should evaluate the advantages of single-use eye drops in the clinical routine.

In summary, we showed that even under maximum efforts to maintain room cleanliness, hygienic provisions, and short duration of use following the recommendations of the Commission for Hospital Hygiene and Infection Prevention at the Robert Koch Institute (KRINKO) there is still a significant amount of contamination of multi-dose eyedrops, which could lead to severe infections like endophthalmitis.

Even if single-dose eyedrops might have disadvantages like higher cost an amounts of plastic waste, by now we would recommend the use of single-dose eyedrops in the peri- and intraoperative context.

## Conclusion

Although the prevalence of contamination was low, the results of this study reveal the possibility of contamination of multi-dose eyedrops even when used by health care professionals in the controlled environment of an operating theatre. Following these results, we recommend the use of single-dose eyedrops in the pre- and intraoperative context.
